# Supplementary material for: Phosphate limitation intensifies negative effects of ocean acidification on globally important nitrogen fixing cyanobacterium
Source: Nat Commun. 2022 Nov 8;13:6730. doi: 10.1038/s41467-022-34586-x (PMC9640675; doi:10.1038/s41467-022-34586-x)
Supplement: Supplementary file 1 — Supplementary Information [file 41467_2022_34586_MOESM1_ESM.pdf]

Supplementary information for

Phosphate limitation intensifies negative effects of ocean acidification on globally important nitrogen fixing cyanobacterium

Futing Zhang[1]#†, Zuozhu Wen[1]#, Shanlin Wang[1], Weiyi Tang[2], Ya-Wei Luo[1], Sven A. Kranz[3], Haizheng Hong[1]*, Dalin Shi[1]*

1. *State Key Laboratory of Marine Environmental Science, Xiamen University, Xiamen, Fujian, PR China*

2. *Department of Geosciences, Princeton University, Princeton, NJ 08544, USA*

3. *Department of Earth, Ocean and Atmospheric Science, Florida State University, Tallahassee, FL 32306, USA*

*To whom correspondence should be addressed. Email: dshi@xmu.edu.cn (D.S.) and honghz@xmu.edu.cn (H.H.)

#These authors contributed equally to this work.

†Present address: The Freddy and Nadine Herrmann Institute of Earth Sciences, Hebrew University of Jerusalem, Jerusalem, Israel

Supplementary Table 1. Carbonate chemistry of the P-limited chemostat culture experiments. Alkalinity and pCO_2 were calculated based on measurements of pH_T and DIC, using carbonic acid dissociation constants of Mehrbach *et al.* [1] that were refit in different functional forms by Dickson and Millero [2]. Errors denote 1 standard deviation (n = 3). Source data are provided as a Source Data file.

**Treatment**	**pH_T**	**DIC (μmol kg^{-1})**	**Alkalinity (μmol kg^{-1})**	**pCO_2 (μatm)**
Ambient	8.01 ± 0.01	1957 ± 4	2234 ± 7	430 ± 5
Acidified	7.81 ± 0.01	2074 ± 2	2254 ± 4	746 ± 8

Supplementary Table 2. POC:POP and PON:POP ratios, C and N_2 fixation rates, cell length and width, and P content in lipid, DNA and RNA of P-limited *T. erythraeum* under ambient and acidified conditions. Errors denote 1 standard deviation (n = 3 biologically independent samples), and asterisks denote significant changes under acidified conditions compared with ambient conditions ($p < 0.01$, two-tailed paired Student's t-test). Source data are provided as a Source Data file.

**Parameters**	**Ambient**	**Acidified**
POC:POP (mol:mol)	332 ± 4	202 ± 14*
PON:POP (mol:mol)	48 ± 2 (20) [a]	31 ± 1* (19) [a]
C fixation rate (mol C mol P^{-1} h^{-1})	5.88 ± 0.44	3.77 ± 0.58*
C fixation rate (mmol C mol C^{-1} h^{-1}) [a]	17.7 ± 1.2	18.9 ± 3.9
N_2 fixation rate (mol N mol P^{-1} h^{-1})	0.31 ± 0.02	0.17 ± 0.03*
N_2 fixation rate (mmol N mol C^{-1} h^{-1})	0.95 ± 0.08 (1.83) [b]	0.85 ± 0.20 (1.50) [b]
N_2 fixation rate (mmol N mol N^{-1} h^{-1}) [a]	6.50 ± 0.23	5.48 ± 0.92
Cell length (µm)	6.59 ± 0.72	5.67 ± 0.50
Cell width (µm)	7.11 ± 0.44	6.40 ± 0.50
Lipid (fmol P $cell^{-1}$)	1.75 ± 0.12	1.27 ± 0.18
DNA (fmol P $cell^{-1}$)	2.6	2.6
RNA (fmol P $cell^{-1}$)	1.45 ± 0.18	1.29 ± 0.31

[a] C-specific C fixation rate and N-specific N_2 fixation rate were calculated by dividing cell number-normalized C and N_2 fixation rates by POC and PON, respectively.

[b] Values in brackets are obtained with *T. erythraeum* cultured under P-replete conditions in a previous study [3].

Supplementary Table 3. Estimated contribution of PO_4^{3-} provided to cellular POP of steady-state P-limited *T. erythraeum* cultured in chemostats under ambient and acidified conditions.

**Treatment**	**Flow rate (mL d^{-1})**	**Stock [PO_4^{3-}] (μM)**	**PO_4^{3-} provided (μmol P d^{-1})**	**Cell density (× 10^4 cell mL^{-1})**	**Cells in outflow (× 10^7 cell d^{-1})**	**POP (fmol $cell^{-1}$)**	**POP in outflow cells (μmol P d^{-1})**	**Contribution of PO_4^{3-} provided to POP (%)**
Ambient-A	300	1.2	0.36	5.31	1.59	23.5	0.374	96
Ambient-B	300	1.2	0.36	4.92	1.48	24.6	0.363	99
Ambient-C	300	1.2	0.36	5.10	1.53	24.8	0.379	95
Acidified-A	300	1.2	0.36	4.03	1.21	31.1	0.376	96
Acidified-B	300	1.2	0.36	4.18	1.25	30.5	0.382	94
Acidified-C	300	1.2	0.36	4.06	1.22	31.7	0.386	93

Supplementary Table 4. Transcriptomic analysis of gene transcription of NAD kinase, proteins involved in Chl*a* synthesis, PSI and ATP synthesis, plasma membrane transporters, enzymes catalyzing polyP synthesis and hydrolysis, and nitrogenase proteins of P-limited *T. erythraeum*. Data are mean of three biological replicates. lfcSE, standard error of the Log_2 Fold change; *p*-value was obtained using the DESeq2 R package and adjusted using the Benhamini and Hochberg's approach; % change = $[2^{Log_2 Fold\ change} - 1] \times 100$. NA: not available.

Functions	Gene name	Log_2 Fold change	lfcSE	*p*-value	% change	Gene description
NAD kinase	*Tery_1770*	-0.417	0.129	0.009	-25	NAD(+) kinase
	Tery_3163	-0.175	0.108	0.348	-11	NAD(+) kinase
Chl*a* synthesis	*hemE*	-0.254	0.110	0.133	-16	uroporphyrinogen decarboxylase
	hemN	-0.461	0.146	0.008	-27	coproporphyrinogen III oxidase
	hemY	-0.284	0.125	0.130	-18	protoporphyrinogen oxidase
	chlI	-0.332	0.149	0.115	-21	magnesium chelatase
	chlB	-0.376	0.137	0.038	-23	light-independent protochlorophyllide reductase subunit B
PSI complex	*psaD*	-0.374	0.090	0.001	-23	photosystem I protein PsaD
	psaE	-0.234	0.094	0.112	-15	photosystem I reaction center subunit IV
	psaI	-0.247	0.110	0.145	-16	photosystem I reaction center subunit VIII
ATP synthesis	*atpH*	-0.293	0.095	0.049	-18	ATP synthase F1, delta subunit
	atpF	-0.283	0.094	0.035	-18	ATP synthase F0, B subunit
	atpG	-0.238	0.110	0.168	-15	ATP synthase subunit b'
	atpE	-0.413	0.127	0.009	-25	ATP synthase F0, C subunit
	atpB	-0.247	0.098	0.099	-16	ATP synthase F0, A subunit
Plasma membrane transporters	*tery_0625*	0.566	0.175	0.002	57	ABC transporter ATP-binding protein
	tery_4204	0.404	0.311	NA	40	K^+ channel, inward rectifier, region 2
	tery_2691	0.433	0.232	0.099	43	polysaccharide export protein
	tery_4422	0.291	0.182	0.261	29	C4-dicarboxylate ABC transporter

polyP synthesis	*ppk1*	-0.127	0.138	0.611	-8	polyphosphate kinase
and hydrolysis	*ppx*	0.081	0.107	0.719	6	exopolyphosphatase
N_2 fixation	*nifB*	0.423	0.113	0.018	34	nitrogenase cofactor biosynthesis protein NifB
	nifH	0.330	0.150	0.121	26	nitrogenase iron protein
	nifW	0.279	0.094	0.038	21	nitrogenase-stabilizing/protective protein NifW

Supplementary Table 5. RT-qPCR analysis of gene transcription of NAD kinase and proteins involved in Chl*a* synthesis and ATP synthesis of P-limited *T. erythraeum*. Data are ratios of copy numbers of the target gene mRNA to *ftsZ* mRNA. Errors denote 1 standard deviation (n = 3 biologically independent samples), and asterisks denote significant changes under acidified conditions compared with ambient conditions ($p < 0.05$, two-tailed paired Student's t-test). % change = [(acidified normalized to ambient condition) – 1] × 100. Source data are provided as a Source Data file.

**Functions**	**Genes**	**Treatment**	**mRNA ratio**	**% Change**	**Gene description**
NAD kinase	*ppnk*	Ambient	0.274 ± 0.022		NAD(+) kinase
		Acidified	0.175 ± 0.037*	-36	
Chl*a* synthesis	*hemN*	Ambient	2.449 ± 0.220		coproporphyrinogen III oxidase
		Acidified	1.925 ± 0.078*	-21	
	hemY	Ambient	0.672 ± 0.019		protoporphyrinogen oxidase
		Acidified	0.460 ± 0.113*	-31	
	chlI	Ambient	0.078 ± 0.018		magnesium chelatase
		Acidified	0.057 ± 0.029	-27	
	chlB	Ambient	0.043 ± 0.008		light-independent protochlorophyllide
		Acidified	0.026 ± 0.015	-40	reductase subunit B
ATP synthesis	*atpH*	Ambient	8.616 ± 0.686		ATP synthase F1, delta subunit
		Acidified	6.763 ± 1.698	-22	
	atpE	Ambient	7.562 ± 0.217		ATP synthase F0, C subunit
		Acidified	5.533 ± 2.354	-27	

Supplementary Table 6. Carbonate chemistry and the average SRP concentration of the incubation experiments conducted at 7 stations in the NSCS. Errors denote SD (n = 3). ND: no data. Source data are provided as a Source Data file.

**Date**	**Station**	**Treatment**	**pH_T**	**DIC (μmol kg^{-1})**	**Alkalinity (μmol kg^{-1})**	**pCO_2 (μatm)**	**SRP (nmol L^{-1})**
May 2016	OA-2	Ambient	7.88 ± 0.01	2025 ± 9	2263 ± 10	560 ± 3	22
		Acidified	7.81 ± 0.00	2073 ± 45	2259 ± 48	750 ± 16	18
July 2017	OA-3	Ambient	7.96 ± 0.02	1959 ± 10	2212 ± 16	496 ± 20	32
		Acidified	7.86 ± 0.02	1957 ± 5	2159 ± 17	628 ± 34	35
July 2017	OA-4	Ambient	7.97 ± 0.01	1959 ± 4	2218 ± 4	483 ± 14	ND
		Acidified	7.83 ± 0.01	1989 ± 4	2177 ± 1	701 ± 19	ND
June 2018	OA-5	Ambient	8.01 ± 0.01	1938 ± 5	2234 ± 5	406 ± 8	16
		Acidified	7.81 ± 0.01	1909 ± 6	2089 ± 9	675 ± 17	16
July 2017	TM-3	Ambient	8.05 ± 0.01	1954 ± 9	2262 ± 6	395 ± 9	30
		Acidified	7.85 ± 0.00	1974 ± 7	2172 ± 8	649 ± 4	21
Aug 2018	TM-4	Ambient	8.08 ± 0.02	1936 ± 15	2224 ± 28	432 ± 21	19
		Acidified	7.85 ± 0.01	2079 ± 17	2260 ± 17	790 ± 15	10
Aug 2018	TM-5	Ambient	8.07 ± 0.00	1922 ± 2	2208 ± 4	427 ± 3	19
		Acidified	7.82 ± 0.00	2070 ± 10	2243 ± 9	819 ± 11	16

Supplementary Table 7. Primers used in sequencing and qPCR analysis of laboratory and/or field experiments.

**Gene name**	**5' primer (5'-3')**	**3' primer (5'-3')**	**Target size (bp)**
**qPCR primers for the laboratory experiments**			
phnD1/ptxA	TGGTGGACAACAACAACGAG	AAGCCCCAATTCTATCTTCTTGAC	158
phnC1/ptxB	GCGATCAAGCTTCTACTTCTAGTC	CATTGGCAACAGCAATAGCAAC	132
phnE1/ptxC	GCTGCTCGCAATACTACTCCT	ATACCCCAGGAAGTGTGCC	139
ptxD	CCCGACTTATTAGCAGCACC	AGCTTTTCCTAATTTTCCCATCCC	189
atpH	GCAGAAGTGACCTCCACAGT	TGATGCCAATACGACGCAGT	199
atpE	GTTGTTGCTGCTGCCTTAGC	GCTTCCATAAATGCCAAGCTCA	158
hemN	ATGGGTGCAGCCAGCTATTT	CCTTGTGCCAAGCGAAATCC	176
hemY	TTGGTGGTGGACTTTTGGCT	GACGAAGACCCACGAGAGAC	201
chlI	CCCCTGGTAGTGGGAAAACC	CCGATGCCGAATGATGAGGA	174
chlB	TGTGGTGGAAACTGCTCGTT	CATTTCCCGCGCCAAAATCT	226
ppnK	TTGCGTTAAACCTGCTTCTGC	CAATAGGGCGGCTCGAAAGA	224
ftsZ	TTAACTCTCTCACGCGCTCC	CACCCACCGTCAAAGAACCT	298
**PCR primers for the field experiments**			
sphX degenerate	ACTACAATTGCTGGRGTYAAG	ATAAGTAACRATAGGATAAGA	~807
ppnK degenerate	AATGTRGGAGGACATTTAGGC	GCCCATAGYAGTTTTTCTCT	~620
**qPCR primers for the field experiments**			
rnpB (Tten)[4]	GAATCTATGAACGCAACGGAAC	ACCAGCAGTGTCGTGAGG	102
rnpB (Tery)[4]	ACCAACCATTGTTCCTTCG	CAAGCCTGCTGGATAACG	199
sphX (Tten)	AGTGGCCAGTGGGTATTGGT	CCAAGGCTGCAGTTTCCAGT	129
sphX (Tery)	CAGTTGGTAGTGGTGCTGG	CTTCAGGTCGGATATACCTGG	179
ppnK (Tten)	CGTTAAACCCGCTTCTGCTG	CCTGCTCCTGTAAAGGCCAA	269
ppnK (Tery)	TTGCGTTAAACCTGCTTCTGC	CAATAGGGCGGCTCGAAAGA	224

Supplementary Table 8. Parameters used to estimate N_2 fixation rate of *Trichodesmium*, UCYN-A, UCYN-B and *Richelia*.

**Diazotrophs**	**Parameters**	**Average ± SD and/or range**	**References**
Trichodesmium	Conversion factor of *nifH* gene copies to cell count (gene copies per cell)	22 ± 33 (1-120)	(5)
		130 ± 239 (1.4-1405)	(6)
	Cell specific N_2 fixation rate (fmol cell^{-1} h^{-1})	21 ± 17 (2-50)	(7)
		12 ± 3 (3-25)	(8)
UCYN-A	Conversion factor of *nifH* gene copies to cell count (gene copies per cell)	1	(9)
	Cell specific N_2 fixation rate (fmol cell^{-1} h^{-1})	10 (1-18)	(10)
Crocosphaera	Conversion factor of *nifH* gene copies to cell count (gene copies per cell)	1	(11)
	Cell specific N_2 fixation rate (fmol cell^{-1} h^{-1})	5 (0.1-135)	(12, 13)
Richelia	Conversion factor of *nifH* gene copies to cell count (gene copies per cell)	542 ± 811	(6)
	Cell specific N_2 fixation rate (fmol cell^{-1} h^{-1})	20.4 (1.15–71.5)	(14)

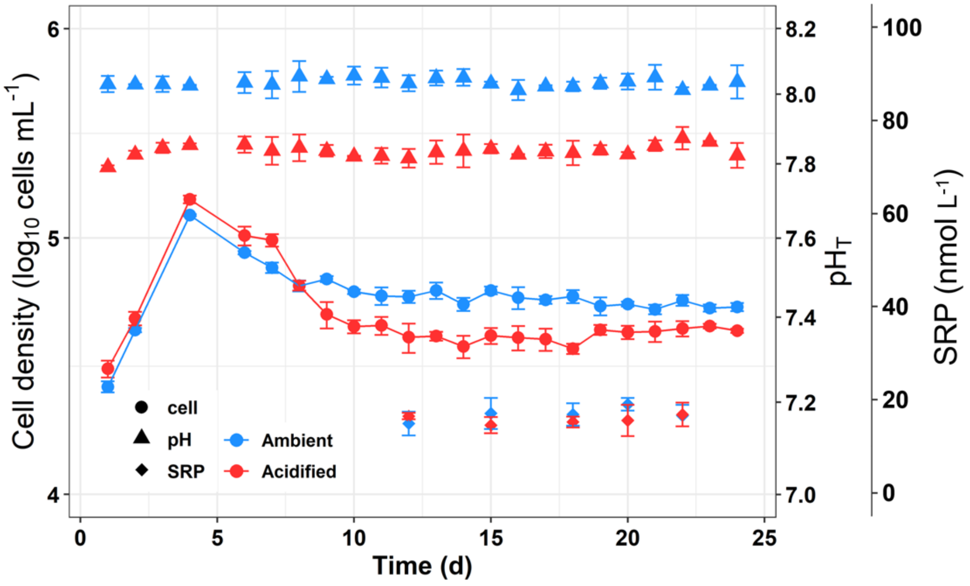

Supplementary Figure 1. Cell density, and medium pH and SRP concentration in the chemostat cultures of P-limited *T. erythraeum* under ambient and acidified conditions. Data are presented as mean values +/– SD (n = 3 biologically independent samples). Source data are provided as a Source Data file.

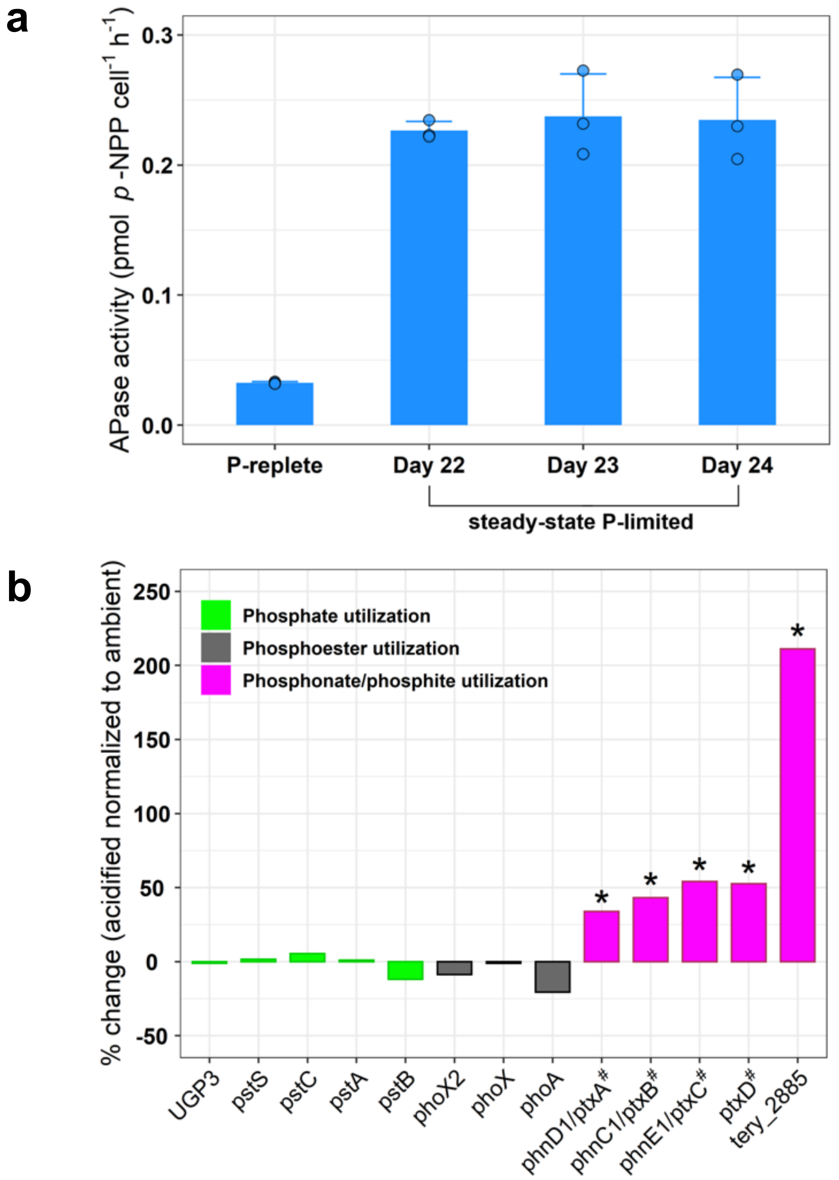

Supplementary Figure 2. (a) Activity of alkaline phosphatase of *T. erythraeum* grown under P-replete conditions (growth rate = 0.54 day^{-1}, samples from a previous study [3]) and under steady-state P-limited conditions in chemostats (growth rate = 0.2 day^{-1}). Data are presented as mean values +/– SD (n = 3 biologically independent samples), and dots are corresponding data points of the replicates. (b) Percentage change (acidified normalized to ambient condition) of gene transcription of proteins involved in the utilization of phosphate, phosphoester, phosphonate and phosphite in P-limited *T. erythraeum*. Asterisks (*) denote significant changes ($p < 0.05$) under

acidified conditions compared with ambient conditions (differential expression was analyzed using the DESeq2 R package and the *p*-values were adjusted using the Benjamini and Hochberg's approach). Pound (#) denote RT-qPCR analysis data and were analyzed using two-tailed paired Student's t-test. Source data are provided as a Source Data file.

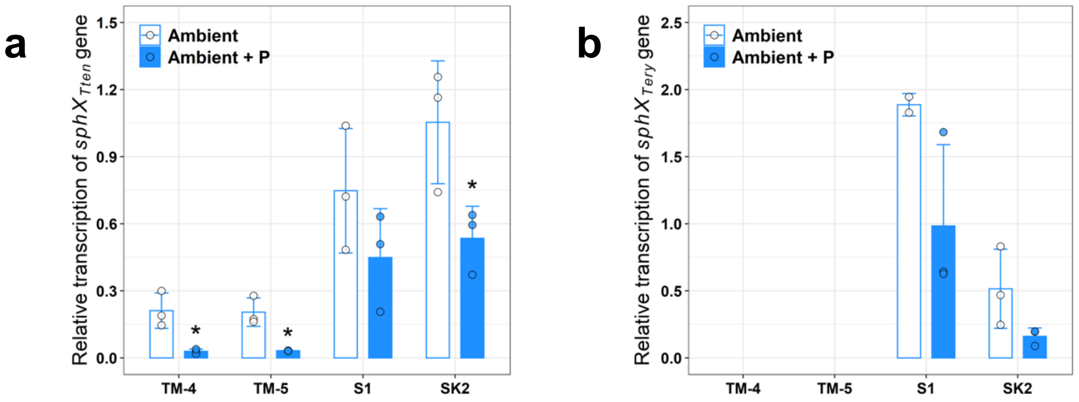

Supplementary Figure 3. Relative transcription of the P-limited biomarker gene *sphX* in *Trichodesmium* clades Tten (a) and Tery (b) collected from stations TM-4, TM-5, S1 and SK2 in the northern South China Sea. Down-regulated *sphX* gene expression in response to PO_4^{3-} addition (100 nM) indicates that the natural *Trichodesmium* populations in the study region were P-limited. Data are presented as mean values +/– SD (n = 2 or 3 biologically independent samples), and dots are corresponding data points of the replicates. Asterisks denote significant changes ($p < 0.05$) with PO_4^{3-} addition (one-tailed paired Student's t-test). Source data are provided as a Source Data file.

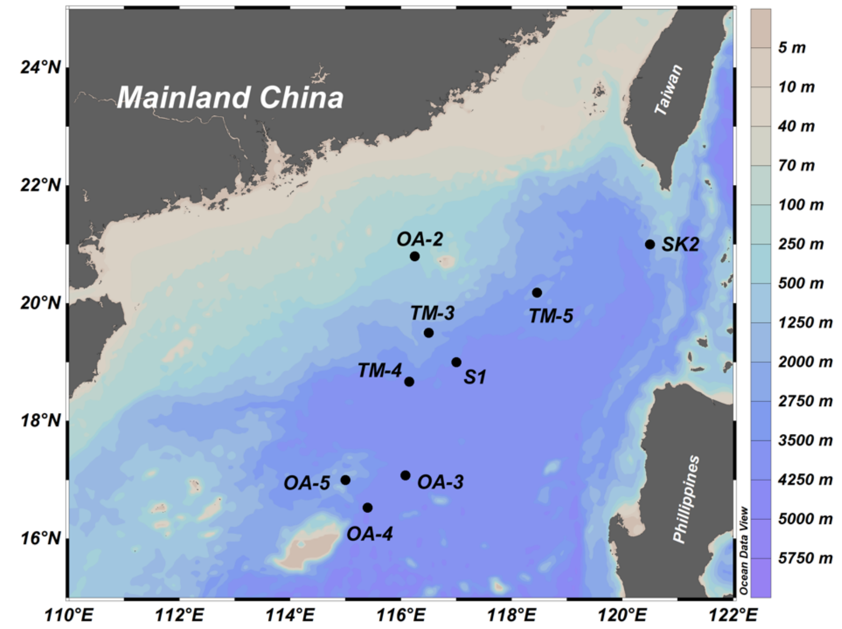

Supplementary Figure 4. Stations where PO_4^{3-} amendment (TM-4, TM-5, S1 and SK2) and ocean acidification (TM-3 to TM-5 and OA-2 to OA-5) experiments were conducted in the northern South China Sea. The map was produced using Ocean Data View 5.

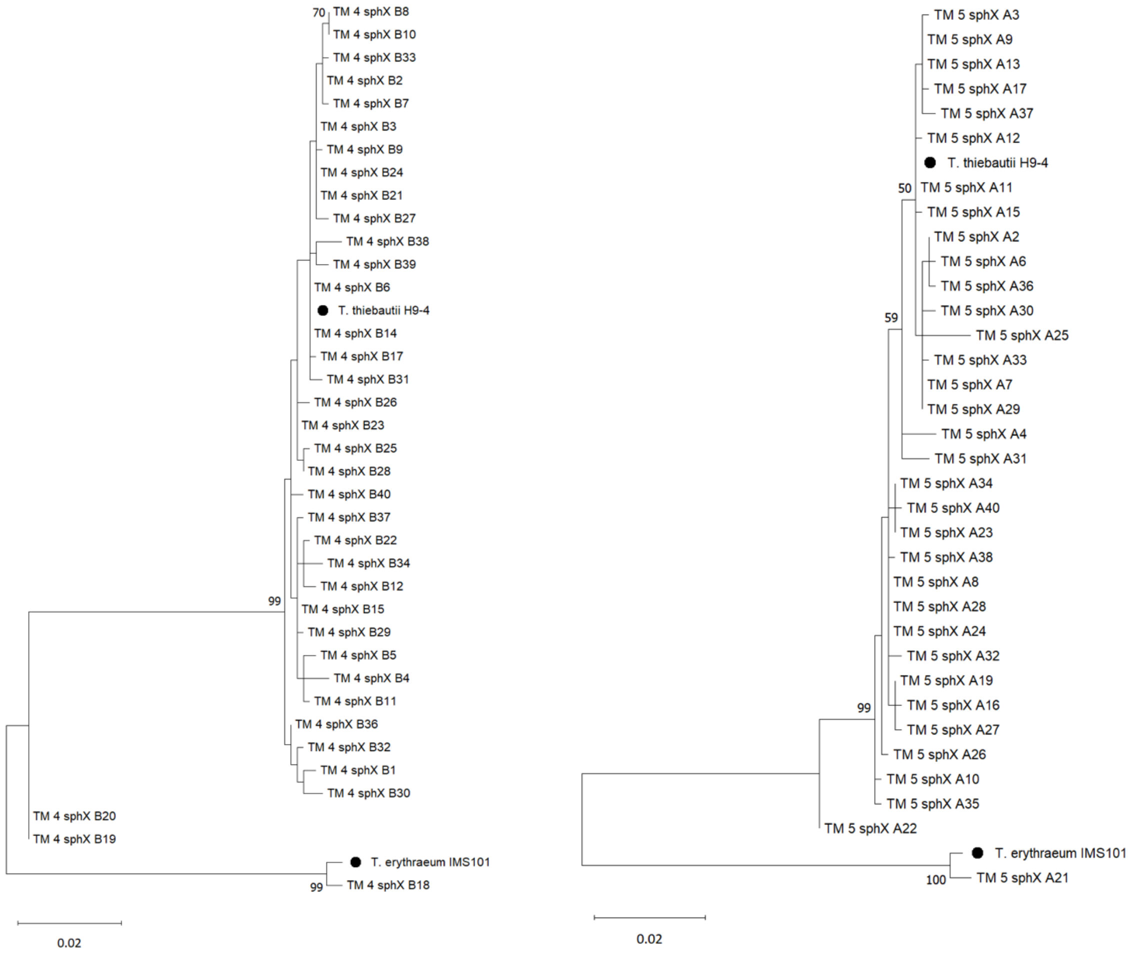

Supplementary Figure 5. Maximum likelihood phylogenetic tree of *Trichodesmium* spp. partial *sphX* nucleotide sequences (807 bp), constructed using clones of *Trichodesmium sphX* PCR products from stations TM-4 and TM-5 and of *T. erythraeum* IMS101 and *T. thiebautii* H9-4 *sphX* sequences in Genbank. Phylogenetic analysis was conducted in MEGA-X with the Hasegawa-Kishino-Yano model (1985) and 1000 bootstrap replicates. Bootstrap values < 50 were removed from the tree. Black dots denote *T. erythraeum* IMS101 (Tery_3534) and *T. thiebautii* H9-4 *sphX* sequences from genome in Genbank.

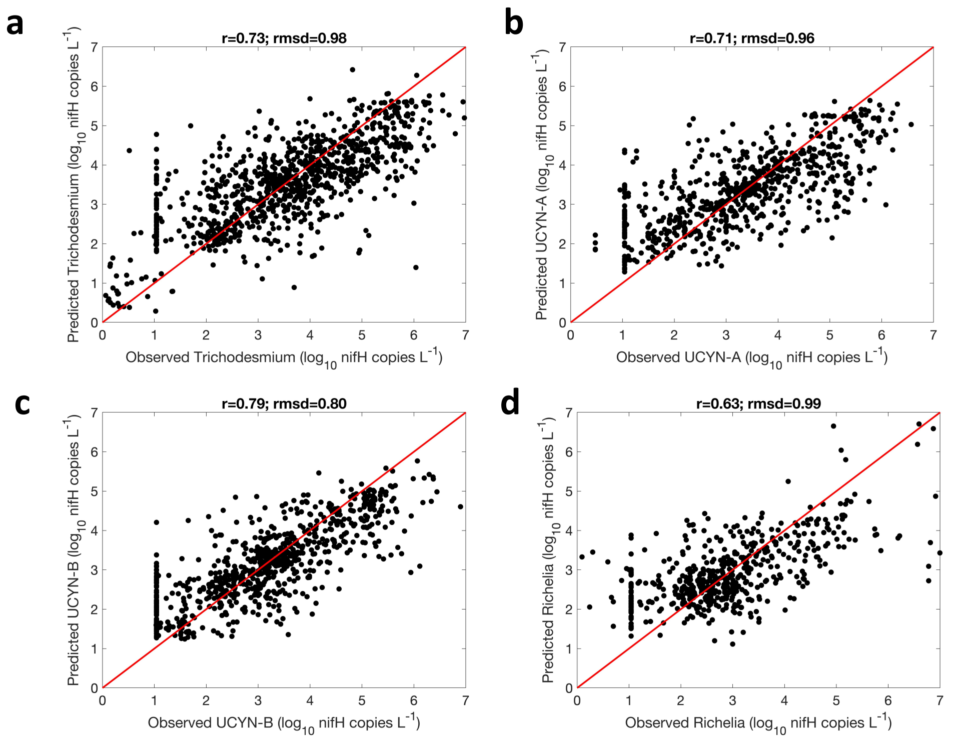

Supplementary Figure 6. Field-observed vs. random-forest models predicted *nifH* gene abundances for (a) *Trichodesmium*, (b) UCYN-A, (c) UCYN-B, and (d) *Richelia* in the out-of-bag test datasets (i.e., data not used for training the random forest models).

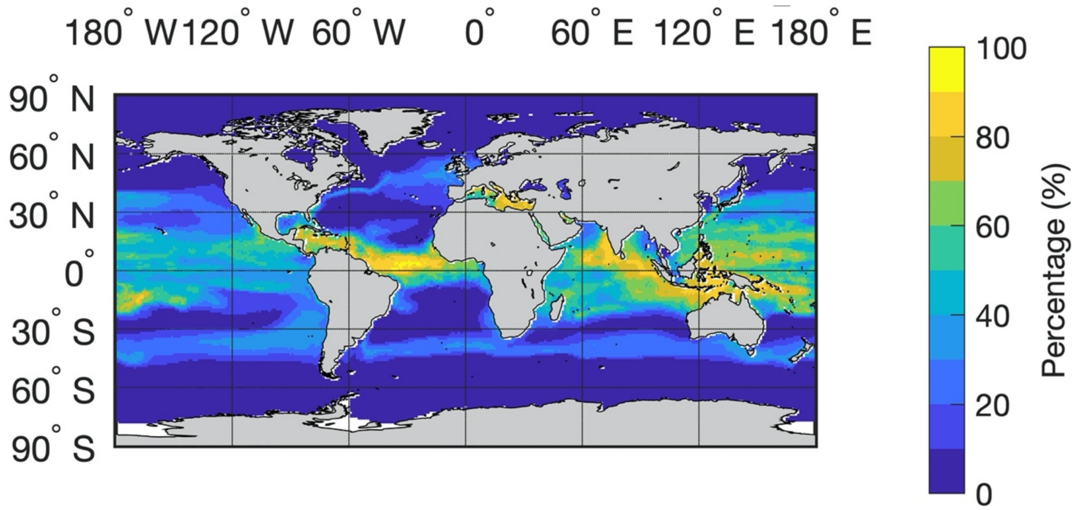

Supplementary Figure 7. Annual average of *Trichodesmium*'s contribution to total N_2 fixation over the top 200 meters in the global ocean. Visualization was conducted using MATLAB_R2021a.

Supplementary Note 1. Growth rates in chemostat cultures.

In a chemostat, the change in biomass dX over an infinitely small-time interval (dt) can be expressed as

$$\frac{dX}{dt} = \mu X - DX = X(\mu - D)$$

where X is cell density (cell L^{-1}), μ is specific growth rate, and D is dilution rate [15,16]. After reaching dynamic equilibrium (steady-state), the cell density remains constant and dX/dt = 0, and thus the specific growth rate equals the dilution rate (μ = D). In our chemostats, the inflow and outflow rates were 0.3 L day^{-1}, and the constant culture volume was maintained at 1.5 L. Therefore, the dilution rates were 0.2 day^{-1} (i.e., inflow rate/constant volume), and at steady-state cells under both ambient and acidified conditions were growing at the same rate of 0.2 day^{-1}.

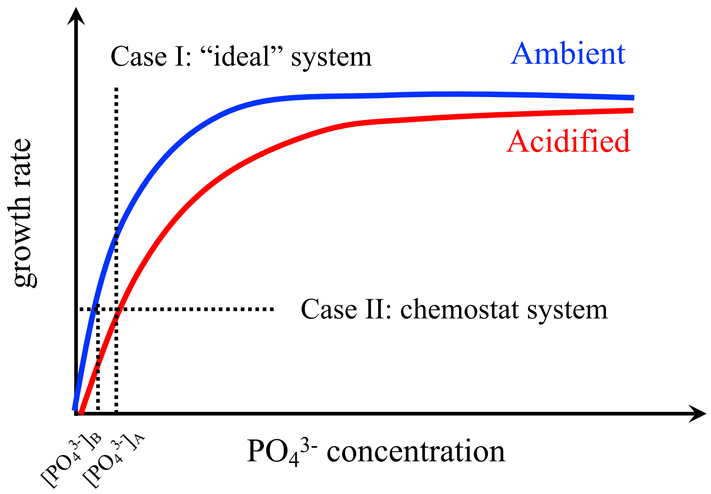

Our study showed that POP of P-limited *T. erythraeum* increased under acidified conditions (Fig. 1b). As illustrated in the conceptual figure above, in an "ideal" steady-state PO_4^{3-}-limited system (Case I), for a given concentration of phosphate (i.e., $[PO_4^{3-}]_A$), a lower growth rate is expected under acidified conditions. However, realistically, such an "ideal" system is difficult to be

experimentally realized. In a chemostat (Case II), such as the one in our study that uses a same dilution rate (i.e., growth rate) for both ambient and acidified treatments, a higher concentration of PO_4^{3-} is needed for cells grown under acidified conditions (i.e., $[PO_4^{3-}]_A > [PO_4^{3-}]_B$). However in chemostats, because of immediate and almost complete consumption of the PO_4^{3-} supplied, both $[PO_4^{3-}]_A$ and $[PO_4^{3-}]_B$ are very low. In the case of our study, $[PO_4^{3-}]_A$ and $[PO_4^{3-}]_B$ were < 20 nM and the difference between them could not be well differentiated by the analytical method we used. It should be noted that the conceptual figure showing here is for illustration purpose, and the two curves (i.e., blue for Ambient and red for Acidified) only represent a possible relationship between growth rate and $[PO_4^{3-}]$ under the two treatment conditions.

Supplementary Methods. The computer code of the numerical model:

```
oaCfix = 0/(10^-7.81 - 10^-8.01);
oapCfix = -0.392/(10^-7.81 - 10^-8.01) ;

oa = -0.184/(10^-7.81 - 10^-8.01);
b=10^-8.01;
oap = -0.101/(10^-7.81 - 10^-8.01) ;

%load input files
Dpre1 = netcdf('b.e11.B20TRC5CNBDRD.f09_g16.001.pop.h.pH_3D.185001-200512.nc');
Dpre2 = netcdf('b.e11.B20TRC5CNBDRD.f09_g16.001.pop.h.diaz_Nfix.185001-200512.nc');
Dpre3 = netcdf('b.e11.B20TRC5CNBDRD.f09_g16.001.pop.h.diaz_Fe_lim.185001-200512.nc');
Dpre4 = netcdf('b.e11.B20TRC5CNBDRD.f09_g16.001.pop.h.diaz_P_lim.185001-200512.nc');
Dpre5 = netcdf('b.e11.B20TRC5CNBDRD.f09_g16.001.pop.h.TEMP.185001-200512.nc');

Drcp1 = netcdf('b.e11.BRCP85C5CNBDRD.f09_g16.001.pop.h.pH_3D.208101-210012.nc');
Drcp2 = netcdf('b.e11.BRCP85C5CNBDRD.f09_g16.001.pop.h.diaz_Nfix.208101-210012.nc');
Drcp3 = netcdf('b.e11.BRCP85C5CNBDRD.f09_g16.001.pop.h.diaz_Fe_lim.208101-
210012.nc');
Drcp4 = netcdf('b.e11.BRCP85C5CNBDRD.f09_g16.001.pop.h.diaz_P_lim.208101-
210012.nc');
Drcp5 = netcdf('b.e11.BRCP85C5CNBDRD.f09_g16.001.pop.h.TEMP.208101-210012.nc');

var1='pH_3D'; var2='diaz_Nfix';

tmp1 = Dpre1{var1}(1573:1812,1:15,:,:); tmp1(tmp1>1e9) = NaN; tmp1(tmp1<0) = 0;
tmp2 = Dpre2{var2}(1573:1812,1:15,:,:); tmp2(tmp2>1e9) = NaN; tmp2(tmp2<0) = 0;
tmp3 = Drcp1{var1}(:,1:15,:,:); tmp3(tmp3>1e9) = NaN; tmp3(tmp3<0) = 0;
tmp4 = Drcp2{var2}(:,1:15,:,:); tmp4(tmp4>1e9) = NaN; tmp4(tmp4<0) = 0;


for mon = 1:12
    Hpre(mon,:,:,:) =   squeeze(nanmean(tmp1(mon:12:240,:,:,:),1)) ;
    Npre(mon,:,:,:) =   squeeze(nanmean(tmp2(mon:12:240,:,:,:),1)) ;
    Hrcp(mon,:,:,:) =   squeeze(nanmean(tmp3(mon:12:240,:,:,:),1)) ;
    Nrcp(mon,:,:,:) =   squeeze(nanmean(tmp4(mon:12:240,:,:,:),1)) ;
end
Hpre =permute(Hpre,[4,3,2,1]);
Npre =permute(Npre,[4,3,2,1]);
```

```
Hrcp =permute(Hrcp,[4,3,2,1]);
Nrcp =permute(Nrcp,[4,3,2,1]);

Hpre = 10.^(-Hpre);
Npre = Npre * 30.4 * 24 * 3600;

Hrcp = 10.^(-Hrcp);
Nrcp = Nrcp * 30.4 * 24 *3600;

tlat = Dpre1{'TLAT'}(:,:);
tlon = Dpre1{'TLONG'}(:,:);
dz=Dpre1{'dz'}(:);dz=dz*0.01; area= permute(Dpre1{'TAREA'}(:,:),[2,1]); area=area*0.0001;
kmt = Dpre1{'KMT'}(:,:); kmt(kmt==0) = NaN; zt=Dpre1{'z_t'}(:);zt=zt*0.01;

Lfepretmp = permute(Dpre3{'diaz_Fe_lim'}(1573:1812,:,:,:),[4 3 2 1]);
Lfepretmp(Lfepretmp>1e9) = NaN;
Lppretmp = permute(Dpre4{'diaz_P_lim'}(1573:1812,:,:,:),[4 3 2 1]); Lppretmp(Lppretmp>1e9)
= NaN;
temppretmp = permute(Dpre5{'TEMP'}(1573:1812,1:15,:,:),[4 3 2 1]);
temppretmp(temppretmp>1e9) = NaN;

Lfercptmp = permute(Drcp3{'diaz_Fe_lim'}(1:240,:,:,:),[4 3 2 1]); Lfercptmp(Lfercptmp>1e9) =
NaN;
Lprcptmp = permute(Drcp4{'diaz_P_lim'}(1:240,:,:,:),[4 3 2 1]); Lprcptmp(Lprcptmp>1e9) =
NaN;
temprcptmp = permute(Drcp5{'TEMP'}(1:240,1:15,:,:),[4 3 2 1]); temprcptmp(temprcptmp>1e9)
= NaN;

for mon =1:12
    Lfepre(:,:,:,mon) = nanmean(Lfepretmp(:,:,:,mon:12:240),4);
    Lppre(:,:,:,mon) = nanmean(Lppretmp(:,:,:,mon:12:240),4);
    temppre(:,:,:,mon) = nanmean(temppretmp(:,:,:,mon:12:240),4);

    Lfercp(:,:,:,mon) = nanmean(Lfercptmp(:,:,:,mon:12:240),4);
    Lprcp(:,:,:,mon) = nanmean(Lprcptmp(:,:,:,mon:12:240),4);
    temprcp(:,:,:,mon) = nanmean(temprcptmp(:,:,:,mon:12:240),4);
end

limpre = Lfepre*0; limrcp = limpre;
Nreplete=0.9;
```

```
for k = 1:15
    for y = 1:384
        for x = 1:320
            for mon=1:12
            if (Lppre(x,y,k,mon) < Lfepre(x,y,k,mon)) && (temppre(x,y,k,mon) >= 15)
                limpre(x,y,k,mon) = 1; % P limiting
            elseif (Lfepre(x,y,k,mon) < Lppre(x,y,k,mon))   && (temppre(x,y,k,mon) >= 15)
                limpre(x,y,k,mon) = 2; % Fe limiting
            elseif (Lppre(x,y,k,mon) > Nreplete)&&(Lfepre(x,y,k,mon) > Nreplete) &&
(temppre(x,y,k,mon) >= 15)
                limpre(x,y,k,mon) = 3; % nutrient replete
            elseif (temppre(x,y,k,mon) < 15)
                limpre(x,y,k,mon) = 0; % no growth
            end
            if (Lprcp(x,y,k,mon) < Lfercp(x,y,k,mon))     && (temprcp(x,y,k,mon) >= 15)
                limrcp(x,y,k,mon) = 1; % P limiting
            elseif (Lfercp(x,y,k,mon) < Lprcp(x,y,k,mon))   && (temprcp(x,y,k,mon) >= 15)
                limrcp(x,y,k,mon) = 2; % Fe limiting
            elseif (Lprcp(x,y,k,mon) > Nreplete) &&   (Lfercp(x,y,k,mon) > Nreplete) &&
(temprcp(x,y,k,mon) >= 15)
                limrcp(x,y,k,mon) = 3; %nutrient replete
            elseif (temprcp(x,y,k,mon) < 15)
                limrcp(x,y,k,mon) = 0; % no growth
            end

            end
        end
    end
end

F1=netcdf('Trichodesmium_N2_Fixation_contribution_v2.nc');
RT=F1{'Tricho_NF_ratio'}(:,:,:,:); latF=F1{'lat'}(:);lonF=F1{'lon'}(:);depF=F1{'depth'}(:);
sf=[141:360,1:140];
RT=RT(:,:,sf,:);RT=permute(RT,[4,3,2,1]);RT=flipud(RT);
for i=1:180
    for j= 1:360
        for mon = 1:12
            RTz(i,j,:,mon)=interp1(depF(1:10),squeeze(RT(i,j,1:10,mon)),zt(1:15),'linear');
        end
    end
end
```

```
maskpop=kmt*0+1;

latitude=[-89.5:1:89.5]';
longitude = [[320.5:1:359.5],[0.5:1:319.5]]';
[ny,nx] = size(RTz(:,:,1,1));
[ncy,ncx] = size(tlat);
[X,Y] =meshgrid(longitude,latitude);

for k=1:15
    for mon =1:12
        tmpdat(:,:)=griddata(X(:,:),Y(:,:),squeeze(RTz(:,:,k,mon)),tlon,tlat);
        RTpop(:,:,k,mon)=tmpdat;
    end
end

RTpop=permute(RTpop,[2,1,3,4]);

SNpre = squeeze(Npre(:,:,1,:))*0;
SNpreT = SNpre;
SNrcp = SNpre;
SNrcpT=SNpre;
SNrcp_oa = SNpre;
SNrcp_oap = SNpre;

for j = 1:384
    for i = 1:320
        for k = 1:15
            for mon = 1:12
            SNpreT(i,j,mon) = SNpreT(i,j,mon) + Npre(i,j,k,mon)* dz(k) ;
            SNpre(i,j,mon) = SNpre(i,j,mon) + Npre(i,j,k,mon)* dz(k)* RTpop(i,j,k,mon) ;
            SNrcpT(i,j,mon) = SNrcpT(i,j,mon) + Nrcp(i,j,k,mon)*dz(k);
            SNrcp(i,j,mon) = SNrcp(i,j,mon) + Nrcp(i,j,k,mon)*dz(k)* RTpop(i,j,k,mon);
             if Hrcp(i,j,k,mon) > b
            SNrcp_oa(i,j,mon) = SNrcp_oa(i,j,mon) + Nrcp(i,j,k,mon)* RTpop(i,j,k,mon) *
max(0,(1+oaCfix*(Hrcp(i,j,k,mon)-b)))* max(0,(1+oa*(Hrcp(i,j,k,mon)-b))) *dz(k) ; % decline
due to acidification
            elseif Hrcp(i,j,k,mon) <= b
            SNrcp_oa(i,j,mon) = SNrcp_oa(i,j,mon) + Nrcp(i,j,k,mon)* RTpop(i,j,k,mon)
*dz(k);
            end
```

```
                if (Hrcp(i,j,k,mon) > b) && (limrcp(i,j,k,mon)==1)
                     SNrcp_oap(i,j,mon) = SNrcp_oap(i,j,mon) + Nrcp(i,j,k,mon) *
RTpop(i,j,k,mon)*max(0,(1+oapCfix*(Hrcp(i,j,k,mon)-b)))*max(0,(1+oap*(Hrcp(i,j,k,mon)-b)))
*dz(k) ; %additional decline due to P limitation
                elseif (Hrcp(i,j,k,mon) > b) && (limrcp(i,j,k,mon)~=1)
                     SNrcp_oap(i,j,mon) = SNrcp_oap(i,j,mon) + Nrcp(i,j,k,mon)   *
RTpop(i,j,k,mon)* max(0,(1+oaCfix*(Hrcp(i,j,k,mon)-b))) * max(0,(1+oa*(Hrcp(i,j,k,mon)-b)))
*dz(k) ; % decline due to acidification
                elseif (Hrcp(i,j,k,mon) <= b)
                     SNrcp_oap(i,j,mon) = SNrcp_oap(i,j,mon) + Nrcp(i,j,k,mon)* RTpop(i,j,k,mon)
*dz(k);
                end
                end

            end
        end
end


TSNpreT = nansum(nansum(nansum(SNpreT,3).*area))/1000 * 14 / 1e12
TSNrcpT = nansum(nansum(nansum(SNrcpT,3).*area)) /1000* 14 / 1e12

TSNpre = nansum(nansum(nansum(SNpre,3).*area))/1000 * 14 / 1e12
TSNrcp = nansum(nansum(nansum(SNrcp,3).*area)) /1000* 14 / 1e12
TSNrcp_oa = nansum(nansum(nansum(SNrcp_oa,3).*area)) /1000* 14 / 1e12
TSNrcp_oap = nansum(nansum(nansum(SNrcp_oap,3).*area)) /1000* 14 / 1e12


color=flipud(hot);
mycolor=[[0.7 0.7 0.7];color(2:64,:)];

tlat=permute(tlat,[2 1]);tlon=permute(tlon,[2 1]);

close all;
figure(11)
POP_pcolor(tlon,tlat,nansum(SNrcp,3));caxis([0 80]);overlay_coastlines
title(['Tricho N fixation (2081-2100), ' num2str(TSNrcp,3) ' TgN/year'])
xlabel('Longitude');ylabel('Latitude');set(gca,'fontsize',16,'fontweight','bold');
c=colorbar;colormap(color);
set(get(c,'ylabel'),'string', 'mmol N/m^2/yr','fontsize',16,'fontweight','bold');
set(gcf,'unit','centimeters','position',[0 1 26 13]);set(0,'defaultfigurecolor','w')
```

```
fg1=getframe(gcf);

figure(12)
POP_pcolor(tlon,tlat,(nansum(SNrcp_oa,3)-nansum(SNrcp,3)));caxis([-20
20]);overlay_coastlines
title(['change in Tricho N fixation, OA - CTRL(2081-2100), ' num2str(TSNrcp_oa-TSNrcp,3) '
TgN/year'])
xlabel('Longitude');ylabel('Latitude');set(gca,'fontsize',16,'fontweight','bold');
c=colorbar;colormap(jet);
set(get(c,'ylabel'),'string', 'mmol N/m^2/yr','fontsize',16,'fontweight','bold');
set(gcf, 'colormap', bluewhitered);set(0,'defaultfigurecolor','w')
set(gcf,'unit','centimeters','position',[0 1 26 13])
fg2=getframe(gcf);

figure(13)
POP_pcolor(tlon,tlat,(nansum(SNrcp_oap,3)-nansum(SNrcp,3)));caxis([-20
20]);overlay_coastlines
title(['change in Tricho N fixation, OA w/ P_l_i_m_i_t - CTRL(2081-2100), '
num2str(TSNrcp_oap-TSNrcp,3) ' TgN/year'])
xlabel('Longitude');ylabel('Latitude');set(gca,'fontsize',16,'fontweight','bold');
c=colorbar;colormap(jet);
set(get(c,'ylabel'),'string', 'mmol N/m^2/yr','fontsize',16,'fontweight','bold');
set(gcf, 'colormap', bluewhitered);set(0,'defaultfigurecolor','w')
set(gcf,'unit','centimeters','position',[0 1 26 13])
fg3=getframe(gcf);

h1 = figure(1);
set(h1,'PaperUnits','inches',...
    'PaperPosition',[.1 .5 8.7 10.5],...
    'DefaultAxesFontName','Helvetica',...
    'DefaultAxesFontSize',9)%
subplot('position',[0.1 0.7 .8 0.3]);
imshow(fg1.cdata);
subplot('position',[0.1 0.4 .8 0.3]);
imshow(fg2.cdata);
subplot('position',[0.1 0.1 .8 0.3]);
imshow(fg3.cdata);
fig1=['map.diaz_Nfix_diff.2000vs2100_OA_20220819.png' ]
print(h1,'-dpng',fig1,'-r600')
```

```
h2 = figure(2);
set(h2,'PaperUnits','inches',...
    'PaperPosition',[.1 .5 8.7 10.5],...
    'DefaultAxesFontName','Helvetica',...
    'DefaultAxesFontSize',9)%
subplot('position',[0.1 0.7 .62 0.26]);
POP_pcolor(tlon,tlat,nansum(SNrcp,3));caxis([0 80]);overlay_coastlines
title(['Tricho N fixation (2081-2100), ' num2str(TSNrcp,3) ' TgN/year'])

subplot('position',[0.1 0.38 .62 0.26]);
POP_pcolor(tlon,tlat,nansum(SNrcp_oa,3));caxis([0 80]);overlay_coastlines
title(['Tricho N fixation, OA (2081-2100), ' num2str(TSNrcp_oa,3) ' TgN/year'])

subplot('position',[0.1 0.05 .62 0.26]);
POP_pcolor(tlon,tlat,nansum(SNrcp_oap,3));caxis([0 80]);overlay_coastlines
title(['Tricho N fixation, OA w/ P limitation (2081-2100), ' num2str(TSNrcp_oap,3) ' TgN/year'])
c=colorbar;colormap(mycolor);set(c,'position',[0.75 0.3 0.04, 0.5])
set(get(c,'ylabel'),'string', 'mmol N/m^2/yr','fontsize',12,'fontweight','bold');

fig2=['map.diaz_Nfix.2000vs2100_OA_20220401.png' ]
print(h2,'-dpng',fig2,'-r600')
```

Supplementary References

1. Mehrbach, C., Culberson, C. H., Hawley, J. E. & Pytkowicx, R. M. Measurement of the apparent dissociation constants of carbonic acid in seawater at atmospheric pressure. *Limnol. Oceanogr.* **18**, 897-907 (1973).
2. Dickson, A. G. & Millero, F. J. A comparison of the equilibrium constants for the dissociation of carbonic acid in seawater media. *Deep Sea Res.* **34**, 1733-1743 (1987).
3. Zhang, F. T. *et al.* Proteomic responses to ocean acidification of the marine diazotroph *Trichodesmium* under iron-replete and iron-limited conditions. *Photosynth. Res.* **142**, 17-34 (2019).
4. Chappell, P. & Webb, E. A molecular assessment of the iron stress response in the two phylogenetic clades of *Trichodesmium*. *Environ. Microbiol.* **12**, 13-27 (2010).
5. Sargent, E. C. *et al*. Evidence for polyploidy in the globally important diazotroph *Trichodesmium*. *FEMS Microbiol. Lett.* **363**, fnw244 (2016).
6. White, A. E., Watkins-Brandt, K. S. & Church, M. J. Temporal variability of *Trichodesmium* spp. and diatom-diazotroph assemblages in the North Pacific Subtropical Gyre. *Front. Mar. Sci.* **5**, 27 (2018).
7. Mulholland, M. R. & Capone, D. G. The nitrogen physiology of the marine N_2-fixing cyanobacteria *Trichodesmium* spp. *Trends Plant Sci.* **5**, 148-153 (2000).
8. Hutchins, D. A. *et al*. CO_2 control of *Trichodesmium* N_2 fixation, photosynthesis, growth rates, and elemental ratios: Implications for past, present, and future ocean biogeochemistry. *Limnol. Oceanogr.* **52**, 1293-1304 (2007).
9. Tripp, H. J. *et al*. Metabolic streamlining in an open-ocean nitrogen-fixing cyanobacterium. *Nature* **464**, 90-94 (2010).
10. Martinez-Perez, C. *et al*. The small unicellular diazotrophic symbiont, UCYN-A, is a key player in the marine nitrogen cycle. *Nat. Microbiol.* **1**, 1-7 (2016).

11. Bench, S. R., Ilikchyan, I. N., Tripp, H. J. & Zehr, J. P. Two strains of *Crocosphaera watsonii* with highly conserved genomes are distinguished by strain-specific features. *Front. Microbiol.* **2**, 261 (2011).
12. Masuda, T. *et al*. Heterogeneous nitrogen fixation rates confer energetic advantage and expanded ecological niche of unicellular diazotroph populations. *Commun. Biol.* **3**, 1-12 (2020).
13. Yang, N. *et al*. Warming iron-limited oceans enhance nitrogen fixation and drive biogeographic specialization of the globally important cyanobacterium *Crocosphaera*. *Front. Mar. Sci.* **8**, 628363 (2021).
14. Foster, R. A. *et al*. Nitrogen fixation and transfer in open ocean diatom-cyanobacterial symbioses. *ISME J.* **5**, 1484-1493 (2011).
15. Rhee, G.Y. Continuous culture algal bioassays for organic pollutants in aquatic ecosystems. *Hydrobiologia* **188**, 247-258 (1989).
16. Borchard, C., Borges, A.V., Händel, N., & Engel, A. Biogeochemical response of *Emiliania huxleyi* (PML B92/11) to elevated CO_2 and temperature under phosphorous limitation: a chemostat study. *J. Exp. Mar. Biol. Ecol.* **410**, 61-71 (2011).
