# Supplementary material for: Phosphate limitation intensifies negative effects of ocean acidification on globally important nitrogen fixing cyanobacterium
Source: Nat Commun. 2022 Nov 8;13:6730. doi: 10.1038/s41467-022-34586-x (PMC9640675; doi:10.1038/s41467-022-34586-x)
Supplement: Supplementary file 3 — Reporting Summary [file 41467_2022_34586_MOESM3_ESM.pdf]

nature portfolio

Corresponding author(s): Dalin Shi, Haizheng Hong

Last updated by author(s): Oct 17, 2022

Reporting Summary

Nature Portfolio wishes to improve the reproducibility of the work that we publish. This form provides structure for consistency and transparency in reporting. For further information on Nature Portfolio policies, see our Editorial Policies and the Editorial Policy Checklist.

Statistics

For all statistical analyses, confirm that the following items are present in the figure legend, table legend, main text, or Methods section.

n/a	Confirmed	
☐	☒	The exact sample size (n) for each experimental group/condition, given as a discrete number and unit of measurement
☐	☒	A statement on whether measurements were taken from distinct samples or whether the same sample was measured repeatedly
☐	☒	The statistical test(s) used AND whether they are one- or two-sided<br>*Only common tests should be described solely by name; describe more complex techniques in the Methods section.*
☐	☒	A description of all covariates tested
☐	☒	A description of any assumptions or corrections, such as tests of normality and adjustment for multiple comparisons
☐	☒	A full description of the statistical parameters including central tendency (e.g. means) or other basic estimates (e.g. regression coefficient) AND variation (e.g. standard deviation) or associated estimates of uncertainty (e.g. confidence intervals)
☐	☒	For null hypothesis testing, the test statistic (e.g. F, t, r) with confidence intervals, effect sizes, degrees of freedom and P value noted<br>*Give P values as exact values whenever suitable.*
☒	☐	For Bayesian analysis, information on the choice of priors and Markov chain Monte Carlo settings
☒	☐	For hierarchical and complex designs, identification of the appropriate level for tests and full reporting of outcomes
☒	☐	Estimates of effect sizes (e.g. Cohen's d, Pearson's r), indicating how they were calculated

Our web collection on statistics for biologists contains articles on many of the points above.

Software and code

Policy information about availability of computer code

Data collection	MATLAB has now been mentioned in the main text and figure legends. Basic climate simulation results were from CESM1-LENS large ensemble community project conducted using CESM1 and CESM2, and further calculations were conducted using MATLAB R2018b
Data analysis	Machine learning toolbox built in MATLAB_R2021a, Bowtie2 version 2.2.3, DESeq2 R package version 1.16, GOSeq version 2.12, KOBAS version 2.0, SigmaPlot 12.5, Mega version 7.0, and ImageJ version 1.8.0, Online Primer-Blast tool, and Ocean Data View 5 were used in the analysis.

For manuscripts utilizing custom algorithms or software that are central to the research but not yet described in published literature, software must be made available to editors and reviewers. We strongly encourage code deposition in a community repository (e.g. GitHub). See the Nature Portfolio guidelines for submitting code & software for further information.

Data

Policy information about availability of data

All manuscripts must include a data availability statement. This statement should provide the following information, where applicable:

- Accession codes, unique identifiers, or web links for publicly available datasets
- A description of any restrictions on data availability
- For clinical datasets or third party data, please ensure that the statement adheres to our policy

All data reported in this study are included in this manuscript or have been deposited in NCBI's Gene Expression Omnibus (GSE181428) and GenBank (MZ749754 – MZ749900), which have been released. The reference genome of T. erythraeum IMS101 can be found in NCBI using the accession number NC_008312.1 (https://www.ncbi.nlm.nih.gov/nuccore/NC_008312.1). Contemporaneous, climatological or modeled environmental predictors are available in World Ocean Atlas 2018 (https://accession.nodc.noaa.gov/NCEI-WOA18).

March 2021

Field-specific reporting

Please select the one below that is the best fit for your research. If you are not sure, read the appropriate sections before making your selection.

☐ Life sciences ☐ Behavioural & social sciences ☒ Ecological, evolutionary & environmental sciences

For a reference copy of the document with all sections, see nature.com/documents/nr-reporting-summary-flat.pdf

Ecological, evolutionary & environmental sciences study design

All studies must disclose on these points even when the disclosure is negative.

Study description

This study mainly focused on the effects of ocean acidification on marine N2 fixer Trichodesmium under phosphorus limitation. We used chemostat cultures in the lab-based experiments, and both lab and field ocean acidification experiments included 2 pH levels of ~7.8 and 8.1. Phosphate amendment experiments included control and P-added (100 nM) treatments. There were 3 replicates for each treatment.

Research sample

For laboratory experiments: we used a single isolate, i.e., Trichodesmium erythraeum strain IMS101, which was most commonly used in laboratory studies. Its lineage is Bacteria, Terrabacteria group, Cyanobacteria/Melainabacteria group, Cyanobacteria, Oscillatoriophycideae, Oscillatoriales, Microcoleaceae, Trichodesmium, Trichodesmium erythraeum, T. erythraeum IMS101 was bought from NCMA (National Center for Marine Algae and Microbiota, US), stably maintained in Aquil-tricho medium at 27 °C and ~80 µmol photons m-2 s-1 (14 h:10 h light-dark cycle) in an AL-41L4 algae chamber (Percival). To mimic phosphorus-limited Trichodesmium in nature, T. erythraeum IMS101 was semi-continuously cultured at 0.5 µM PO43- under ambient or acidified condition for more than one year, and was subsequently grown for about one month in PO43--limited chemostats.

For filed experiments: water samples were collected from the northern South China Sea, a typical oligotrophic region with surface nitrate concentration at nanomolar level that receives sufficient dust depositions from the land. These conditions are favorable for Trichodesmiun to flourish.

Sampling strategy

For laboratory experiments: to measure the pHT of media, accurate 1 mL fresh cultures were placed into a cuvette, immediately mixed well with Thymol Blue (20 µmol L-1). To measure DIC, around 50 mL fresh cultures were transferred into a 50-mL brown glass bottle, immediately added HgCl2 (0.05%, v/v). Samples for Chla measurement were vacuum filtered onto 3 µm polycarbonate membrane filters (Millipore), placed into 1.5-mL tubes with 1 mL 90% ethanol. Samples (5 mL each) for Cell density and the average cell length and width, were fixed by Lugol's solution, then placed on Sedgwick-Rafter counting chambers. Samples for POC, PON and POP, were vacuum filtered onto pre-combusted 25 mm GF/F filters (Whatman). To measure C uptake rate, accurate 50 mL fresh cultures were placed in an acid-washed Nalgene bottle, added 100 µM NaH14CO3. To measure N2 fixation rate, accurate 15 mL fresh cultures were sealed in an acid-washed 60-mL serum bottle. To measure AP activity, accurate 5 mL fresh cultures were placed in an acid-washed 10-mL glass bottles, added 250 µL of 10 mM pNPP, 675 µL of Tris-glycine buffer (50 mM, pH 8.5) and 67.5 µL of 1 mM MgCl2. Samples for SRP were vacuum filtered onto 25 mm GF/F filters (Whatman). Samples for PolyP, Cellular ATP and RNA extraction, were vacuum filtered onto 3 µm polycarbonate membrane filters (Millipore), flash frozen in liquid nitrogen and stored at -80 °C until analysis. Samples for metabolites of NAD(H), NADP(H), and Glu, were vacuum filtered onto 3 µm polycarbonate membrane filters (Millipore), rapidly suspended in -80 °C precooled methanol-water (60%, v/v) mixture. According to most literature, previous studies, and high reproducibility of preliminary experiments of our study, 3 biologically independent replicates were sufficient to produce high reproducible results.

For field experiments: water samples were collected near the surface using ether a trace-metal-clean towed fish system or a regular Sea-bird CTD-General Oceanic rosette sampler with GO-Flo bottles. 2-20 L bottles were used to collect the waters for ocean acidification experiments. The (sub)sampled volumes from the surface water and the bioassay incubations were decided according to phytoplankton biomass that we measured. In general, 3 biologically independent replicates were applied, which were sufficient to produce high reproducible results.

Data collection

For Laboratory experiments: the pHT of media, Chla concentration, POP, SRP and AP activity were separately measured by a spectrophotometer (UV-1800 Spectrophotometer, Shimadzu, Japan), and further calculated and organized by standard curves, samples biomass or volume, and/or incubation time. DIC of media was analyzed by acidification and subsequent quantification of released CO2 with a CO2 analyzer (LI 7000, Apollo SciTech). Alkalinity and pCO2 were calculated using the CO2Sys program. Cell density and cell length and width were determined using ImageJ software. Photographs of Trichodesmium for ImageJ were taken using a camera (Canon DS126281, Japan) connected with an inverted microscope (Olympus CKX41, Japan). POC and PON were measured on a PerkinElmer Series II CHNS/O Analyzer 2400 (PerkinElmer, USA), and further calculated by standard curves and samples biomass. C uptake rates were calculated based on samples biomass, incubation time and the radioactivity of 14C measured by a Tri-Carb 2800TR Liquid Scintillation Analyzer (PerkinElmer, USA). Rates of N2 fixation were calculated based on samples volume, incubation time and the production of acetylene measured by a gas chromatograph (Shimadzu GC-8A, Shimadzu, Japan). PolyP and Cellular ATP were separately measured by a PerkinElmer EnSpire® Multimode Plate Reader (PerkinElmer, USA), and further calculated and organized by standard curves, samples biomass or incubation time. Metabolites of NAD(H), NADP(H), and Glu were measured on an Agilent 1290–6490 UPLC-triple quadrupole mass spectrometry system (Agilent Technologies, Palo Alto, California, USA), and further calculated by samples biomass and standard curves. All qPCR reactions were carried out on a fluorescent quantitative instrument CFX 96 TOUCH (Bio-Rad Laboratories), the expression of target genes was normalized to the abundance of the housekeeping gene FtsZ transcript. Chla concentration, pH of media, cell density, SRP, cellular ATP, PolyP, RNA extraction, qPCR, C uptake and N2 fixation rates, and elemental composition (POC, PON and POP) were performed by Futing Zhang with help from group members in Dalin Shi's lab. Intracellular metabolites (NAD(H), NADP(H), and Glu) were performed by Haizheng Hong and Futing Zhang. Sampling for all measurements was performed at the last day of the chemostat culturing by group members in Dalin Shi's lab. RNA-seq was performed in Novogene (https://en.novogene.com/).

For field experiments: the rate-related data (e.g N2 fixation rate) were measured using a Thermo Finnigan Delta V plus isotope ratio mass spectrometer. The gene abundance and transcription (Quantiative qPCR analysis) were measured using CFX 96 TOUCH (Bio-

	Red, singapore). All data were recorded by Zuozhu Wen and Futing Zhang.
Timing and spatial scale	For laboratory experiments: chemostat cultures were carried out from April 10, 2016 (day 1) to May 3, 2016 (day 24). To exclude the influences of diel rhythm on results, all samples were always collected at the middle of photoperiod. To monitor the development of T. erythraeum IMS101 and the carbonate chemistry of media, Chla concentration and media pH were measured daily, except for day 3 and day 5 when the dilution rate of chemostat was not stable. To avoid the disturbances of sampling and meanwhile have enough data to represent the steady-state of chemostat, different samples were collected at different regular intervals. Samples for cell density were collected at day 15, 17, 19, 21 and 23. Samples for DRP were collected at day 12, 15, 18, 20 and 22. And AP activity was measured at day 22, 23 and 24. Cellular ATP, C uptake and N2 fixation rates, PolyP, intracellular metabolites (NAD(H), NADP(H), and Glu), elemental composition (POC, PON and POP) and RNA extraction, these samples requiring more biomass were collected only at the end of the chemostat culturing (May 3, 2016).<br>For field experiments: samples were collected during 3 cruises in 2016-2018 (2016.05; 2017.07; 2018.08). All sampling sites were within an area with latitude of 16-21ºN and longitude of 115-118.5 ºE. Timing and location of sample collection were based on the season, geographic location and the nutrient concentration. For example, we aim to conduct sampling during warm seasons, and at stations with environmental conditions (e.g. low nutrient concentration) that are favorable for Trichodesmium growth.
Data exclusions	No data were excluded from the analysis in this study.
Reproducibility	For lab-based experiments: All measurements were referenced to previously published protocols. Each measurement was always conducted in the same manner. We performed the same experimental procedure three times over a year and the results were highly robust.<br>For filed experiments: The negative effect of ocean acidification on Trichodesmium dominated diazotroph community was reproduced at 6 of the 7 independent stations.
Randomization	For lab-based experiments: Trichodesmium trichomes were randomly distributed in Nalgene® magnetic culture vessels hence subsamples were collected randomly.<br>For filed experiments: The water samples collected in situ were mixed then randomly allocated into different groups for manipulation experiments.
Blinding	We were subjected to collecting a tremendous amount of samples from multiple treatments in both laboratory and field experiments. Therefore, we had to label treatments and samples clearly and accurately, especially for field experiments. Hence it was not feasible for us to conduct sample collection and follow sample measurements blindly. We made very effort to avoid bias on experimental setups, samplings, measurements, and data analysis by randomization.

Did the study involve field work? ☒ Yes ☐ No

Field work, collection and transport

Field conditions	Sea surface waters were collected to set up the experiments. Sea surface temperature of our sampling location ranges from 29.3-30.5 °C, surface SPR concentration during our sampling is 10-35nM. Samplings were conducted either during the daytime or night. No rainfall happened during the sampling at any station.
Location	The samples were collected from the surface of northern South China Sea basin (water depth ~5m, latitude: 16-21°N, longitude 115-118.5°E).
Access & import/export	The water samples were incubated on-board then frozen at -20 or -80 degree and transported back to laboratory for chemical and molecular analyses. Our permits were obtained from Xiamen University on Jun. 15, 2016
Disturbance	Nearly no disturbance was caused by the study since only 2-20L seawaters were collected from each site.

Reporting for specific materials, systems and methods

We require information from authors about some types of materials, experimental systems and methods used in many studies. Here, indicate whether each material, system or method listed is relevant to your study. If you are not sure if a list item applies to your research, read the appropriate section before selecting a response.

Materials & experimental systems

n/a	Involved in the study	
☒	☐	Antibodies
☒	☐	Eukaryotic cell lines
☒	☐	Palaeontology and archaeology
☒	☐	Animals and other organisms
☒	☐	Human research participants
☒	☐	Clinical data
☒	☐	Dual use research of concern

Methods

n/a	Involved in the study	
☒	☐	ChIP-seq
☒	☐	Flow cytometry
☒	☐	MRI-based neuroimaging
